# Mammographic density and inter-observer variability of pathologic evaluation of core biopsies among women with mammographic abnormalities

**DOI:** 10.1186/1471-2407-12-554

**Published:** 2012-11-24

**Authors:** Pietro Trocchi, Giske Ursin, Oliver Kuss, Kathrin Ruschke, Andrea Schmidt-Pokrzywniak, Hans-Jürgen Holzhausen, Thomas Löning, Christoph Thomssen, Werner Böcker, Alexander Kluttig, Andreas Stang

**Affiliations:** 1 Institute of Clinical Epidemiology, Martin-Luther-University Halle-Wittenberg, Halle (Saale), Magdeburger Str. 8, Halle (Saale), 06097, Germany; 2 Cancer Registry of Norway, Postboks 5313 Majorstuen, Oslo, 0304, Norway; 3 Department of Nutrition, University of Oslo, Oslo, Norway; 4 Department of Preventive Medicine, University of Southern California, Los Angeles, CA, USA; 5 Institute of Medical Epidemiology, Biometry and Informatics, Martin-Luther-University Halle-Wittenberg, Halle (Saale), Germany; 6 Department of Radiology, Martin-Luther-University Halle-Wittenberg, Ernst-Grube-Str. 40, Halle (Saale), 06097, Germany; 7 Institute of Pathology, Martin-Luther-University Halle-Wittenberg, Magdeburger Str. 14, Halle (Saale), 06097, Germany; 8 Albertinen-Pathology Hamburg, Fangdieckstr. 75 a, Hamburg, 22547, Germany; 9 Department of Gynaecology, Martin-Luther-University Halle-Wittenberg, Ernst-Grube-Str. 40, Halle (Saale), 06097, Germany; 10 Reference Center for Gynaeco- and Mammapathology, Fangdieckstr. 75 a, Hamburg, 22547, Germany

**Keywords:** Biopsy, Breast diseases, Mammographic density, Observer variation

## Abstract

**Background:**

As high percentage of mammographic densities complicates the assessment of imaging findings, mammographic density may influence the histopathological evaluation of core-biopsies of the breast. We measured the influence of mammographic density on the inter-observer variability of histopathological findings of breast biopsies.

**Methods:**

Histological slides of 695 women who underwent core biopsies of the breast at University of Halle between 2006 and 2008 were evaluated in a blinded fashion by two pathologists using the five levels of the B-categorization scheme (B1-B5). To quantify mammographic density, we used a computer-based threshold method (Madena). We calculated observed and chance-corrected agreements (weighted kappa) and 95% confidence intervals (95% CI) according to four categories of mammographic density (<10%, 10<25%, 25<50%, ≥50%).

**Results:**

The weighted kappa decreased monotonically from 89.6% (95% CI: 85.8%, 93.3%) among women with less than 10% of mammographic density to 80.4% (95% CI: 69.9%, 90.9%) for women with more than 50% of mammographic density, respectively. Results of a kappa regression analysis showed that agreement of pathologists on clinically relevant categories (B1-B2 versus B3-B5) decreased with mammographic density.

**Conclusions:**

Mammographic density is a relevant modifier of the agreement between pathologists who assess breast biopsies using the B-categorization scheme. The influence of mammographic density on the inter-observer variability can be explained to some extent by varying prevalences of histological entities across B categories that have typically different inter-observer agreement. Women with high mammographic density are at higher risk of inter-observer variability compared to women with low mammographic density and should possibly undergo a second pathology review.

## Background

The breast is composed of a mixture of fibroglandular tissue that appears bright on a mammogram (radio dense) and fatty tissue that appears radiological transparent (radiolucent). The radiological appearance of fibroglandular breast tissue is quite similar to that of breast lesions. Therefore, high mammographic density may mask breast lesions and reduce the probability that cancerous lesions are detected. Mammographic density is a well described indicator of increased risk of breast cancer and may be related to the degree of diagnostic certainty [1,2].

Several studies have shown that mammographic density is negatively associated with the performances of breast cancer screening. In a United Kingdom’s study Carney et al. reported that the sensitivity of mammography decreased from 87% in women with almost entirely fatty breasts to 62.9% in women with extremely dense breasts [3]. Britton et al. observed a reduction of mammographic sensitivity with increasing mammographic density according to a four category density classification [4]. As high mammographic density complicates the assessment of the imaging findings and the histopathological diagnostic should be performed in correspondence with the mammographic findings, a high mammographic density may be associated with higher inter-observer variability between pathologists who evaluate biopsy material. To date, no studies have reported results about the influence of mammographic density on the reliability of histopathological findings of breast biopsies that are currently classified by the B-categorization according to the National Coordinating Group for Breast Screening Pathology [5]. The B-categorization includes five reporting categories. Categories B1-B2 usually do not require further invasive diagnostic workup unless biopsies classified as B1 were uninterpretable or unrepresentative of the breast lesion according to the imaging and clinical findings. Categories B3-B5 usually require further invasive workup. The European guidelines recommend to check that the histological findings correlate with the mammographic findings in order to interpret correctly the histological material of core biopsies of the breast and before to define the diagnosis [6].

To assess mammographic density patterns, both qualitative and quantitative approaches have been used. Wolfe described first in 1976 a qualitative method to assess mammographic density using a classification of the breast tissue based on the description of four parenchyma-patterns of the breast that defines four level of mammographic density [7]. More recently, the American College of Radiology (ACR) developed in 1993 the Breast Imaging Reporting and Data System (BIRADS), a classification of breast tissue density that is also based on four categories of mammographic density (I: mostly fatty, II: fibroglandular, III: heterogeneous dense, IV: extremely dense) with the aim of increasing the uniformity in interpretations of mammographic findings [8]. However, according to the results of a study of Kerlikowske et al. in 1998, there is considerably variability in interpreting mammographic density by applying this method [9]. Ciatto et al. reported in 2005 that the interobserver agreement on assessing breast densities according to BIRADS was “moderate” (Kappa=0.54) [10]. In our study, we used a computer-assisted method of assessing mammographic density [11].

The aim of this study was to assess the influence of mammographic density estimated by a quantitative scale on the inter-observer variability of the pathologic evaluations of core-biopsies of the breast.

## Methods

The design and first results of the study were described in detail previously [12,13]. The Diagnosis Optimisation Study (DIOS) was approved by the institutional review board of the Medical Faculty of the Martin-Luther-University of Halle-Wittenberg.

In brief, we recruited women who underwent core biopsies of the breast at the Department of Radiology between April 2006 and August 2008. All women provided an informed consent for participation in the study. As women of the organized mammography screening program who were biopsied by ultrasound guidance were not referred to the university they were not eligible for this study. Overall, 30 women were not eligible for several reasons: core biopsy technically impossible (N=22), microcalcification of the skin (N=3), lack of sufficient command of the German language (N=3), and other reasons (N=2). A total of 98 women refused to participate. An additional 29 women only partially agreed to participate. Partial agreement included histopathological assessment by the pathologists but excluded the questionnaire-based assessment of breast cancer risk factors, including weight and height of the women. Women below the age of 18 were excluded from the study. For 66 women, mammographic density assessment was technical impossible and, therefore, these women were excluded from the analysis, giving a final study population which included 695 women.

As in our previous publication [13], we defined four groups of women who were referred to the university for further work-up of imaging abnormalities of the breast (“referral groups”): women referred from the organized mammography screening program in Halle for stereotactic core biopsy (“screening”), women with a history of breast cancer with abnormal follow-up breast images (“history of breast cancer”), women with clinical symptoms of the breast (“clinical symptoms”) and women without symptoms with abnormal imaging findings outside the screening program (“only images”). The available pre-biopsy imaging findings including mammographic density were recorded from the radiologists according to the four categories of the Breast Imaging Reporting and Data System (BIRADS) of the American College of Radiology (I, II, III and IV). The level of suspicion of the lesions were documented on a scale of 0 to 6 including “additional imaging evaluation recommended” (BIRADS-code 0), “negative (no abnormal lesions)” (BIRADS-code 1), “benign lesions” (BIRADS-code 2), “probably benign lesions” (BIRADS-code 3), “suspicious abnormality” (BIRADS-code 4), “highly suggestive of cancer” (BIRADS-code 5), and “biopsy-proven malignancy” (BIRADS-code 6). Biopsies were performed by either stereotactic-, ultrasound- or magnetic resonance-guided vacuum-assisted methods or by the ultrasound-guided automated gun method. The choice of imaging technique for the biopsies depended on the detection method of the breast abnormality, and the size and type of lesion. Histological slides of the biopsies were assessed by overall three pathologists. First, the local pathologist at the Martin-Luther-University of Halle (Pathologist 1, H.J.H.) reviewed the haematoxylin and eosin-stained (HE) slides from paraffin-embedded blocks of the biopsy specimens. If necessary to confirm the diagnosis or to define receptor status, immunhistochemical (IHC) staining was performed. Second, in order to implement a reference standard, a second pathologist (W.B.) reviewed the material and made the reference diagnosis. Disagreements between the local and reference pathologist were resolved by consensus based on a telephone conference. Third, the pathologist from the Albertinen-Pathology Hamburg (Pathologist 2, T.L.) evaluated in a blinded fashion the identical set of slides that had been assessed by the pathologist of the University of Halle. To estimate the interobserver agreement between the histopathological evaluations of the biopsies, we compared the assessments of pathologist 1 and 2, since the reference pathologist was not fully blinded against the diagnosis of pathologist 1 and had the opportunity to perform additional IHC staining to confirm the diagnosis.

The radiologists who undertook the core biopsy provided information about age, localization of biopsy, number of biopsy cores, microcalcification, and a description of the focus. Furthermore, for women with microcalcification, the pathologists received the X-ray images of the paraffin blocks containing the biopsy specimen. The result of the core biopsy was interpreted in relation to the imaging findings (i.e. mammography, ultrasound or magnetic resonance image depending on the radiologic technique). The histopathological findings were documented on a standardized case report form that included both the traditional diagnosis of the histological findings and the B-categories (B1: normal or uninterpretable, B2: benign, B3: benign but of uncertain biological potential, B4: suspicious of malignancy, and B5: malignant including in-situ and invasive cancer. Category B5 is further subcategorized into B5a (in situ), B5b (invasive), B5c (uncertain whether it is in-situ or invasive), and B5d (other malignancies). This scheme is recommended by the European Guidelines for quality assurance in mammography screening and among women with symptomatic breast lesions with the aim of standardizing histopathological reporting [5,6]. After histopathological evaluation, it was documented, whether the histopathological findings correlated with the imaging findings.

To assess mammographic density on a quantitative scale, we used the Madena computer-based threshold method, a method that has been validated and described previously [11,14]. Mammographic images were digitized using a scanner (VIDAR’s DiagnosticPro Advantage film digitizer) and were then viewed on a computer screen. With few exceptions, we read the craniocaudal mammogram selected from the unaffected breast. In brief the assessment was carried out as follows: The image was imported to Madena and the total area of the breast was outlined by a reader trained by an experienced reader (G.U.) using a special computerized outlining tool and the software estimated the total number of pixels in the breast. This measurement corresponds to the total breast area. Second, the reader defined a region of interest in the breast that contains dense tissue, with exception of the areas corresponding to the pectoralis muscle, prominent veins, fibrous strands and other light artefacts. The reader then used a tinting tool to make dense areas within the region of interest that have a threshold intensity of grey at or above a pixel value of X and below a pixel value of 255. The computer program assigns a pixel value within a range between zero for the darkest shade of the image and 255 for the lightest shade of the image. The reader searches for the threshold that enables best assessment of mammographic densities. The total number of tinted pixels within the region of interest represents the absolute area of mammographic density. The percent of mammographic density equals to the ratio of the dense area to the total area of the breast in the mammogram. The readers were blinded to all subject characteristics.

### Statistical analysis

**Figure 1:**
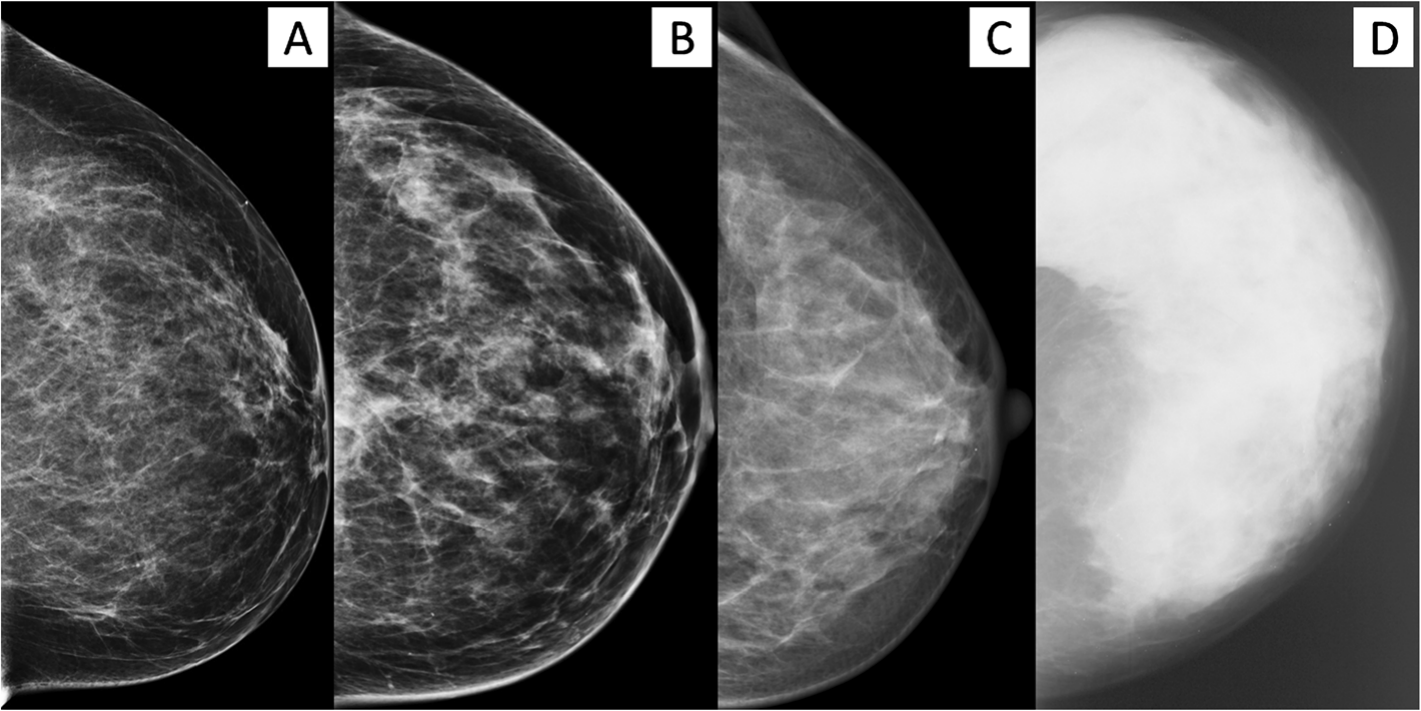
**Categories of percentage of mammographic density according to the computer-assisted assessment method (A: <10%; B: 10<25%; C: 25<50%; D: ≥50%)**.

To quantify mammographic density, we measured both the absolute area of the breast that appeared dense and the proportion of mammographic image representing radiographically dense breast. We categorized the percentage of mammographic density into one of four classes (Figure 1: <10%, 10-<25%, 25-<50%, ≥50%).

To evaluate the B-categorization agreement between the pathologists, we estimated observed and chance-corrected agreements (weighted kappa) and 95% confidence intervals (95% CI). We estimated measures of agreement using the five levels of the B-categorization scheme (B1-B5). Clinically relevant categories which imply the subsequent use of different therapeutic strategies are B1-B2 (usually require no further work-up) and B3-B5 (usually require further invasive work-up). To assess the influence of breast density on agreement of the pathologists (as measured as agreement on B1-B2 versus B3-B5), we ran a kappa regression model [15]. This model allows the estimated kappa coefficient to vary for different values of breast density while additionally adjusting for covariates. To communicate results from this model we estimated kappa values for the range of observed values of mammographic density with their 95% confidence intervals. Furthermore, we assessed the association between percentage of mammographic density and potential determinants of radiological mammographic density, including age and body mass index (BMI).

180 out of 695 women underwent more than one core biopsy of the breast. To avoid a statistical dependency between these biopsies, we only analysed data of the first biopsies of these women. All statistical analyses were performed with SAS 9.2.

## Results

The analysis included 695 women. Table 1 shows the characteristics of the study subjects. The mean age at biopsy was 56.1 years, the mean BMI was 26.6 kg/m^2^. Overall, 55% of the women underwent a mammographic-guided core biopsy, 28% underwent a sonographic-guided biopsy and 17% underwent a MRI-guided core biopsy. The median percentage of breast tissue with densities was 18% (mean percent density: 23%). The median area of breast with mammographic densities was 154 cm^2^ (mean area: 301 cm^2^). The prevalence of diagnoses categorized as B5 according to the reference standard was overall 30.4% and decreased progressively from 38.8% for women with less than 10% density to 12.8% for women with more than 50% (Table 2).

**Table 1:** **Characteristics of women who underwent core biopsy at Martin**-**Luther**-**University of Halle **(**Saale**), **Germany**, **between April 2006 and August 2008 **(**N**=**695**)

**Characteristic**	**N**	**%**
Age (Years)		
<40	39	5.6
40-49	165	23.7
50-59	215	30.9
60-69	214	30.8
70+	62	8.9
BMI (kg/m^2^)		
<21	98	14.1
21-24.9	228	32.8
25-29.9	218	31.4
30+	151	21.8
Biopsy technique		
Stereotactic	381	54.8
Ultrasound-guided	194	27.9
MRI-guided	120	17.3
Referral group		
Screening	83	11.9
History of breast cancer	90	13.0
Clinical symptoms	128	18.4
Only images	394	56.7
BIRADS		
2-3	75	10.8
4	346	49.8
5	204	29.3
6	70	10.1
Radiological findings		
Radiological focus	396	57.0
Microcalcification	346	49.8
Mammographic breast density (ACR)		
almost entirely fatty	58	8.4
fibroglandular	282	41.0
heterogenous dense	283	41.1
extremely dense	65	9.5
Mammographic breast density (Computer-based assessment)		
<10%	242	34.8
10% to <25%	181	26.0
25% to <50%	186	26.8
≥50%	86	12.4

**Table 2:** **Frequency distribution of B**-**categorized biopsies according to the reference diagnosis **(**Reference standard**-**B**)

**Density (%)**	**Women**	**B1**		**B2**		**B3**		**B5**	
		**N**	**%**	**N**	**%**	**N**	**%**	**N**	**%**
<10%	242	20	8.3	108	44.6	20	8.3	94	38.8
10 to <25%	181	14	7.7	86	47.5	28	15.5	53	29.3
25 to <50%	186	19	10.2	83	44.6	31	16.7	53	28.5
≥50%	86	5	5.8	65	75.6	5	5.8	11	12.8
Overall	695	58	8.4	342	49.2	84	12.1	211	30.4

**Figure 2:**
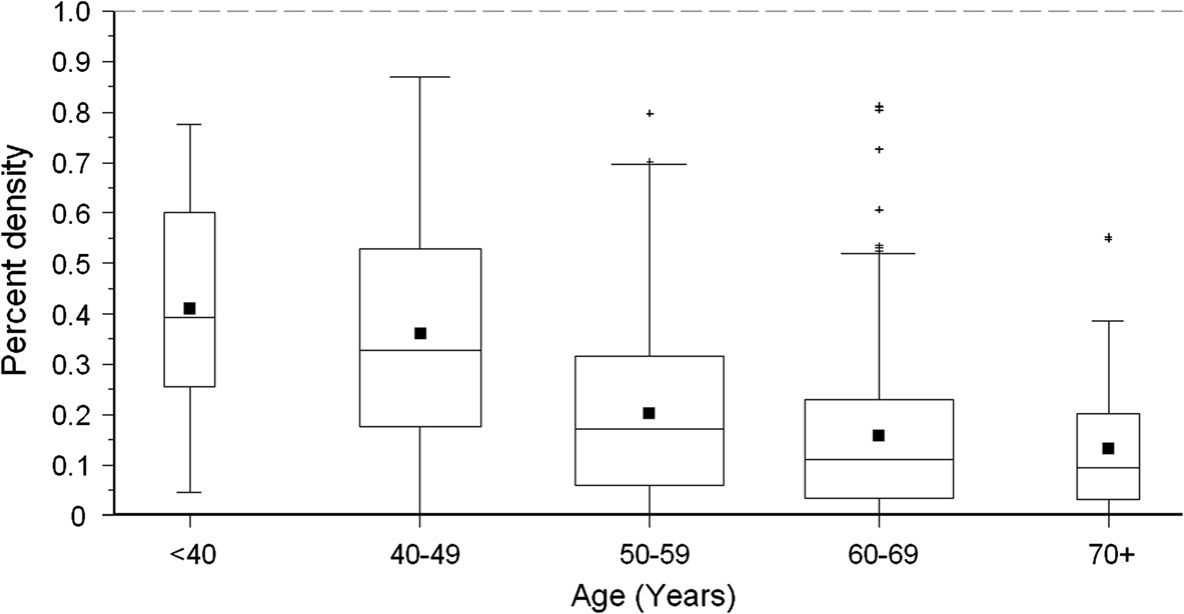
**Distribution of percent densities among the 695 study subjects of the DIOS-Study 2006**–**2008 according to age group.** Box width varies with age group size. Whiskers indicate observations between the lower and upper fence [i.e. 1.5*interquartile range (IQR) below 25^th^ percentile and 1.5*IQR above 75^th^ percentile]. Plus symbols indicate observations outside the fences. Filled squares indicate mean percent density values. Horizontal lines within the boxes indicate the median percent density values.

**Figure 3:**
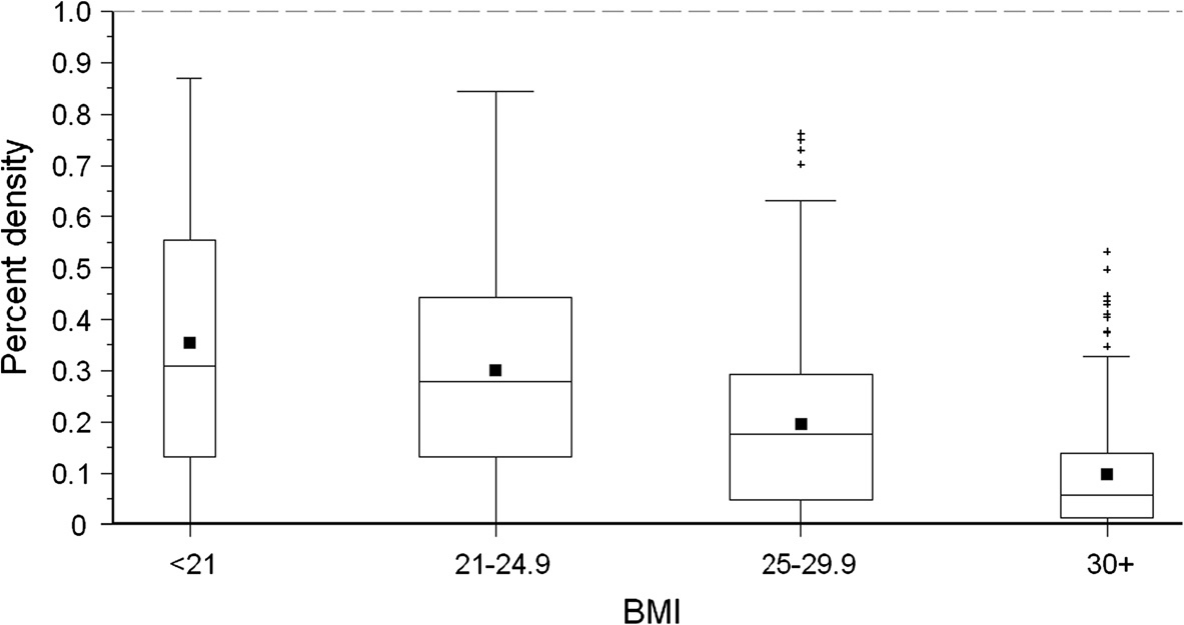
**Distribution of percent densities among the 695 study subjects of the DIOS-Study 2006–2008 according to body mass index (BMI).** Box width varies with age-group size. Whiskers indicate observations between the lower and upper fence [i.e. 1.5*interquartile range (IQR) below 25^th^ percentile and 1.5*IQR above 75^th^ percentile]. Plus symbols indicate observations outside the fences. Filled squares indicate mean percent density values. Horizontal lines within the boxes indicate the median percent density values.

The comparison of the results of the mammographic density assessment based on the quantitative computer-assisted method and those based on the qualitative method developed from the American College of Radiology (ACR) revealed that higher percentage of mammographic density was associated with higher ACR category of mammographic density (Table 3). The median percentage of mammographic density ranged from 1% for women with breasts that were “mostly fatty” (ACR I) to 50% for women with ACR category “dense” (ACR IV). Figures 2 and 3 show that the percent density was negatively associated with age and BMI. The median percent density decreased from 39% respectively for women aged less than 40 years to 10% respectively for women aged more than 70 years. The median percent density decreased from 31% among women with BMI lower than 21 kg/m^2^ to 6% among women with BMI higher than 30 kg/m^2^. About three out of four women (76%) in the lowest group of percent density (lower than 10% density) had BMI higher than 25 kg/m^2^.

**Table 3:** **Percentage of mammographic density of the study subjects by categories of mammographic density based on the Breast Imaging Reporting and Data System **(**BIRADS**) **of the American College of Radiology**

**ACR category**	**N**	**Percent density**	
		**Mean**	**Median**
Mostly fatty (ACR I)	58	3	1
Fibroglandular (ACR II)	282	11	8
Heterogeneously dense (ACR III)	283	33	31
Dense (ACR IV)	65	52	50
Missing evaluations	7	22	13
Overall	695	23	18

Overall, the observed and the chance-corrected agreement based on the five levels of the B-categorization scheme were 85.5% (95% CI: 82.6%, 88.0%) and 87.9% (95% CI: 85.5%, 90.3%), respectively. Table 4 shows that the inter-observer agreement was inversely associated with the percentage of mammographic density. The weighted kappa decreased monotonically from 89.6% (95% CI: 85.8%, 93.3%) among women with less than 10% of mammographic density to 80.4% (95% CI: 69.9%, 90.9%) for women with a mammographic density of 50% or more, which corresponds to an estimated absolute difference of 9.2% points (95% CI: −2.0%, 20.3%). The observed agreement decreased from 87.6% (95% CI: 82.8%, 91.5%) among women in the lowest category to 82.6% (95% CI: 72.9%, 89.9%) for women in the highest category of percent density. The median percent of mammographic density among women for whom there was agreement on the B-category of the diagnosis was lower by 4% points compared to subjects for whom there was no agreement between the pathologists (22% vs. 18%).

**Table 4:** **Percent of observed agreement and chance**-**corrected agreement **(**weighted kappa values**) **of histopathological evaluation **(**five level B**-**categorization**) **according to percent of mammographic density**

**Percent density**	**N**	**Agreement (95% CI)**	
		**Observed**	**Kappa**
<10%	242	87.6 (82.8-91.5)	89.6 (85.8-93.3)
10% to <25%	181	86.2 (80.3-90.9)	88.2 (83.5-92.8)
25% to <50%	186	83.3 (77.2-88.4)	86.4 (81.6-91.2)
≥50%	86	82.6 (72.9-89.9)	80.4 (69.9-90.9)
Overall	695	85.5 (82.6-88.0)	87.9 (85.5-90.3)

**Figure 4:**
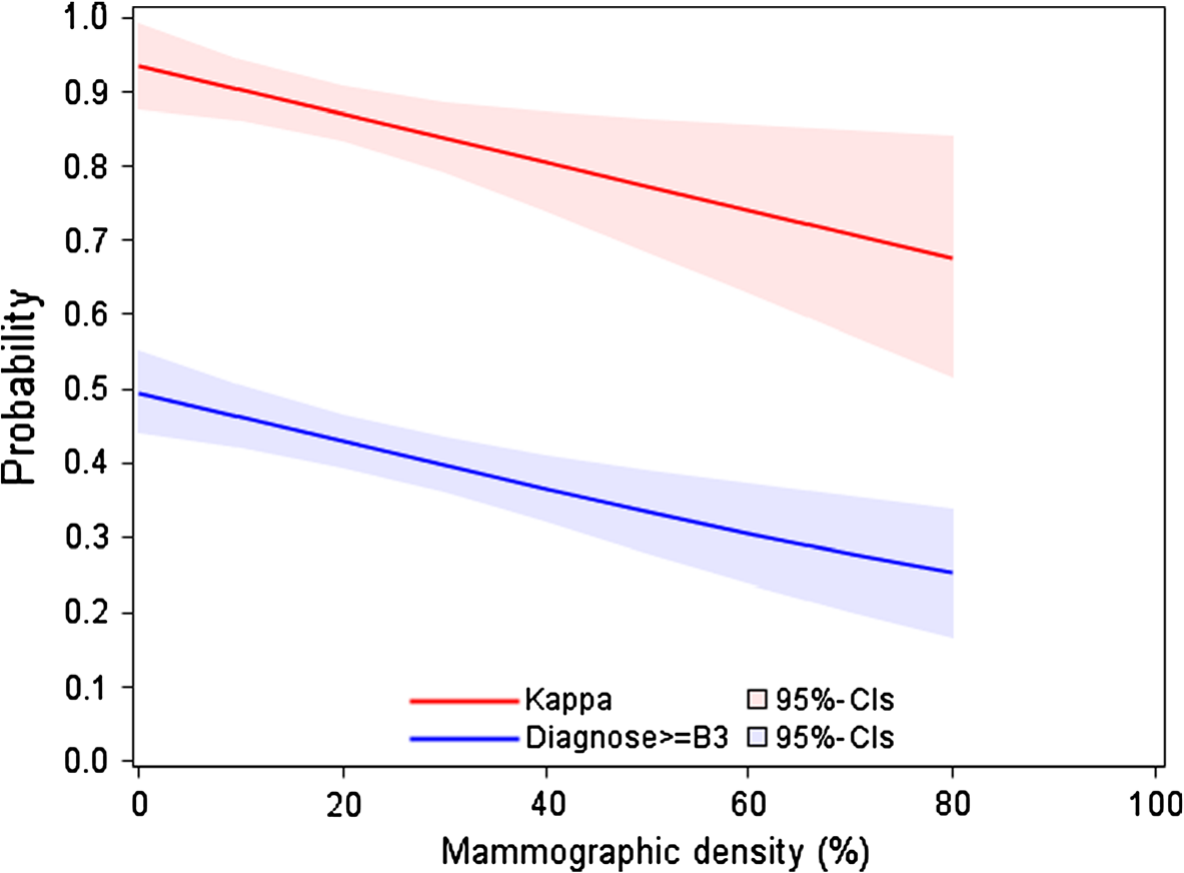
**Chance-corrected agreement (weighted kappa values) of histopathological evaluation and probability of B3-B5 according to percent of mammographic density.** Note: Categories B3-B5 usually require further invasive work-up.

Figure 4 shows that agreement of the pathologists on B1-B2 versus B3-B5 decreased with mammographic density. To explore the reasons of this decrease, we calculated the frequency of B2-B3 disagreements according to level of mammographic density. We found that the number of B2-B3 disagreements increased with mammographic density more than the disagreements overall (data not shown). Adjustment for mammographic findings and level of suspicion of the lesions (BIRADS-code) did not change these results.

Among 34 study participants, the histological findings did not correlate with imaging findings. These women had a higher percent-density (median 27%) and area of mammographic density (median 343 cm^2^) compared to women for whom the correlation between histologic and imaging findings was observed (median percent-density: 17%, median area of density: 140 cm^2^).

## Discussion

Our study shows that a higher percentage of mammographic density is associated with lower inter-observer agreement of the histopathological evaluation of core biopsies of the breast using the B-categorization. The weighted kappa for women with very low percentage of mammographic density (less than 10%) was 9.2% points (95% CI: -2.0%; 20.3%) higher than the weighted kappa for women with mammographic density of 50% or more. Our findings suggest that women with radiological breast densities are at higher risk of disagreement and, if possible, should undergo more than one pathology review.

Mammographic density complicates the evaluation of the imaging findings because the radiological appearance of the fibroglandular breast tissue and that of the mammographic abnormalities can be very similar. Due to a masking effect of cancers by dense breast tissue, a higher mammographic density decreases the sensitivity of mammographic diagnostic, which is to say that increases the risk that a carcinoma will be obscured on the mammogram. We found that the prevalence of carcinomas, according to the reference standard, decreased with mammographic density. Therefore, as carcinomas of the breast are typically lesions with a high inter-observer agreement, the association between high mammographic density and low inter-observer agreement can be partially explained by the lower prevalence of B5-categories among women with higher percentage of mammographic density. The results of the regression analysis imply that the probability of agreement on categories that usually require no further work-up (B1-B2) versus categories that usually require invasive work-up (B3-B5) was modified by the percentage of mammographic density. Although based on few data, our findings suggest that this decrease is due to an increasing frequency of disagreements about the neighbouring B categories B2 and B3 that encompass histological lesions for which the choice of the appropriate therapeutic strategy is most problematic. As expected, women for whom the histological lesions did not correlate to the mammographic abnormalities had a substantially higher percent density in comparison to women for whom the histologic and the imaging findings were correlated (median: 27% vs. 17%). To perform the histopathological assessment of the breast biopsy, according to the European Guidelines, the radiological findings are compared with the histological appearance of biopsy specimens in order to verify the representativeness of the biopsy. Therefore, as high mammographic density complicates the task of the pathologist to ensure that histological findings are representative for imaging findings, high mammographic density decreases the probability to observe inter-observer agreement between pathologists who review biopsy material.

We provide evidence that the percentage of the breast area with densities decreases progressively with age and BMI of the women. These results are consistent with those of several other studies [1,16]. Therefore, age and BMI may be considered as modifiers of the level of interobserver agreement of evaluations of breast biopsies, as these factors influence mammographic density. Finally, we observed a positive association between the results of the mammographic density assessment based on the classification system developed by the American College of Radiology (BIRADS) and those based on the computer-assisted method used in this study. Several studies on the assessment of ACR-based density categorization have shown that the inter- and intra-observer variability of interpretations of mammographic densities is only moderate [9,10]. Computer-assisted methods provide a measure of the projected area of dense tissue on a continuous scale and could represent a less subjective alternative to categorical methods of quantifying breast densities.

Our study has some limitations. First, the histological slides were reviewed in a blinded fashion by only two pathologists experienced breast pathologists. However, certified breast cancer centers in Germany and other countries request experienced pathologists who are certified in the evaluation of core biopsies of the breast. Second, as the computer-based method to assess mammography density measurement is not completely automatically done, some measurement error of the mammography density may have occurred. To minimize this error, a well-trained experienced reader (G.U.) who measured several thousand mammograms before this study did all readings in a centralized way for this study.

## Conclusions

Mammographic density is a relevant modifier of the agreement between pathologists who assess breast biopsies using the B-categorization scheme. The influence of mammographic density on the inter-observer variability can be explained to some extent by the prevalence of histological entities that have different inter-observer agreement. Our findings suggest that women with radiological breast densities are at higher risk of disagreement compared to women with low mammographic density and, if possible, should undergo more than one pathology review.

## Abbreviations

CI: Confidence interval; ACR: American College of Radiology; BIRADS: Breast Imaging Reporting and Data System; DIOS: Diagnosis Optimisation Study; HE: Haematoxylin and eosin; IHC: Immunhistochemical; BMI: Body mass index.

## Competing interests

The authors declare that they have no competing interests.

## Authors’ contributions

PT contributed to the design of the study, collected the data, carried out the statistical analysis and wrote the manuscript. GU carried out the mammographic density measurements and revised the manuscript. AS was responsible for the design of the study, data analysis and interpretation of the findings and revised the manuscript. OK and AK made substantial contributions to the analysis and interpretation of the data and revised the manuscript. ASP and CT were involved in reviewing the manuscript for important intellectual content. KR contributed important clinical information. HJH, TL and WB assessed the histological slides of the biopsies, made substantial contributions to the interpretation of the data and revised the manuscript. All authors read and approved the final manuscript.

## Pre-publication history

The pre-publication history for this paper can be accessed here:

http://www.biomedcentral.com/1471-2407/12/554/prepub
